# Role of berberine in anti-bacterial as a high-affinity LPS antagonist binding to TLR4/MD-2 receptor

**DOI:** 10.1186/1472-6882-14-89

**Published:** 2014-03-06

**Authors:** Ming Chu, Ran Ding, Zheng-yun Chu, Ming-bo Zhang, Xiao-yan Liu, Shao-hua Xie, Yan-jun Zhai, Yue-dan Wang

**Affiliations:** 1 Department of Immunology, School of Basic Medical Sciences, Peking University, No.38, Xueyuan Road, Haidian District, Beijing 100191, China; 2 Pharmacy Departments, Liao Ning University of Traditional Chinese Medicine, Liao Ning 116600, China

## Abstract

**Background:**

Berberine is an isoquinoline alkaloid mainly extracted from *Rhizoma Coptidis* and has been shown to possess a potent inhibitory activity against bacterial. However, the role of berberine in anti-bacterial action has not been extensively studied.

**Methods:**

The animal model was established to investigate the effects of berberine on bacterial and LPS infection. Docking analysis, Molecular dynamics simulations and Real-time RT-PCR analysis was adopted to investigate the molecular mechanism.

**Results:**

Treatment with 40 mg/kg berberine significantly increased the survival rate of mice challenged with *Salmonella typhimurium* (LT2), but berberine show no effects in bacteriostasis. Further study indicated that treatment with 0.20 g/kg berberine markedly increased the survival rate of mice challenged with 2 EU/ml bacterial endotoxin (LPS) and postpone the death time of the dead mice. Moreover, pretreatment with 0.05 g/kg berberine significantly lower the increasing temperature of rabbits challenged with LPS. The studies of molecular mechanism demonstrated that Berberine was able to bind to the TLR4/MD-2 receptor, and presented higher affinity in comparison with LPS. Furthermore, berberine could significantly suppressed the increasing expression of NF-κB, IL-6, TNFα, and IFNβ in the RAW264.7 challenged with LPS.

**Conclusion:**

Berberine can act as a LPS antagonist and block the LPS/TLR4 signaling from the sourse, resulting in the anti-bacterial action.

## Background

Berberine is an isoquinoline alkaloid extracted from variety species of plants such as *Rhizoma Coptidis*[1]. It has been widely used for treatment of skin inflammation, diabetes, liver disease [2-4]. More importantly, berberine has been used in the treatment of enteritis [5,6] for thousands of years in China, and presents the advantages of shorter treatment course, faster currative effect, little side effect, and milder tolerance. However, the role of berberine in antibacterial action has not been extensively studied. Since the process of bacterial infection and the pathogenic mechanisms of bacterial are complicated, it is hard to define the role of berberine in anti-bacterial action without an in-depth study. Enteritis is most commonly caused by the ingestion of substances contaminated with pathogenic microorganisms, such as Salmonella and Escherichia [7]. The symtoms of enteritis include abdominal pain, cramping, dehydration, diarrhea and fever, which are mainly caused by the bacterial endotoxin infection.

Endotoxin is considered to be a toxin kept “within” the bacterial cell and to be released only after destruction of the bacterial cell wall. Nowadays, endotoxin is used synonymously with the term LPS, which is a major constituent of the outer cell membrane of Gram-negative bacteria [8,9]. The key effects of LPS on vertebrates are mediated by their interaction with specific receptors on immune cells such as monocytes, macrophages, dendric cells, and others. LPS consists of a hydrophobic anchor, known as lipid A, a repeating O-antigen polysaccharide, and an inner core oligosaccharide [10,11]. Many of the immune activating abilities of LPS can be attributed to the lipid A unit, which binds to the toll-like receptor 4 (TLR4), and activates the host defence effector system by rapidly triggering pro-inflammatory processes [12-15]. TLR4 alone does not directly bind LPS and requires its coreceptor myeloid differentiation protein (MD-2) which is associated with the extracellular domain of TLR4 and is indispensable for LPS recognition since MD-2 has a unique hydrophobic cavity which can directly bind the lipid A unit [16,17]. The combination between LPS and TLR4/MD-2 receptor complex can cause intense innate immune response [18-20]. After binding LPS, TLR4 undergoes oligomerization and recruits its downstream adaptors via interactions with the TIR (Toll-interleukin-1 receptor) domains. MyD88 (myeloid differentiation primaryresponse gene 88) is one of the five TIR domain-containing adaptor proteins. The TLR4 signaling has been divided into MyD88-dependent and TRIF-dependent path-way. MyD88 activates the downstream transcription factors AP-1, IRF-5 and NF-κB. These transcription factors induce expression of proinflammatory cytokine genes, and thus a series of proinflammatory cytokines (TNF-α, IFN-γ, IL-1, IL-6) are produced through the MyD88-dependent pathway [13,21,22]. Currently, LPS is the most important factor in induction of IL-1 and TNF [23]. These cytokines can regulate the immune responses via the activation of various transcription factors within the fever-controlling region of the hypothalamus to increase the body temperature [24]. Meanwhile, inflammatory reactions induced by LPS can also cause diarrhea [25]. Furthermore, according to the recent evidences, TLR4-mediated elevations in the expression of these cytokines in astrocytes in the central nervous system (CNS) and glia cells can initiate and propagate several debilitating disease states such as sepsis and chronic pain [26-29]. One study demonstrated that berberine inhibits LPS-stimulated myocardial TNF-alpha production, impairs calcium cycling, and improves LPS-induced contractile dysfunction in intact heart within cardiomyocytes exposed to LPS adopted [30]. Another study which adopted LPS-stimulated macrophages demonstrated that berberine could inhibit COX-II activity [31]. Other studies have indicated that Berberine could attenuate tissue injury of the lungs and intestine in mice challenged with LPS and could suppress the activation of NF-κB [32-35]. The above mentioned studies seem to indicate that berberine may block LPS signaling and have some preventitive effects on LPS-induced injury. However, previous research did not clearly identify the stage at which berberine blocks the LPS inflammatory signal.

The crystal structure of human TLR4/MD-2/Ra-LPS complex [36] has been reported and may interact with berberine since it has a large hydrophobic surface, which is likely to compete with LPS in binding to its specific TLR4/MD-2 receptor, thus blocking the LPS/TLR4 signaling. Based on the previous in vitro and animal work, and on our hypothesis that berberine may block the LPS signaling by binding to the TLR4/MD-2 receptor, berberine may present alleviative effects on LPS-induced disease states. In the present study, we first confirmed the anti-bacterial effect. Afterward, we investigated the therapeutic effects of berberine on LPS-induced diseases, thereby providing the evidence supporting the need for the study of anti-endotoxin mechanism. Furthermore, we explored the molecular mechanisms of the action of berberine.

## Methods

### Materials

Berberine was provided by Liaoning University of Traditional Chinese Medicine. Endotoxin was provided by National Institutes for Food and Drug Control. (titer: 9000 EU/amp).

### Bacterial strains

Wild type *S.typhimurium* LT2 was purchased from ATCC (Catalog number: 15277). LT2 was grown in Luria-Bertani (LB) medium at 37°C with shaking at 200 rpm. When appropriate, the medium was supplemented with 10 μg of tetracycline per ml medium or 10 μg of chloramphenicol.

### Cell culture and treatment

Murine macrophage-like cells (RAW264.7) were purchased from ATCC and cultured in Dulbecco’s modified Eagle’s medium (Mediatech Inc., Herndon, VA, USA) supplemented with 100 μg/ml of penicillin/streptomycin and 10% heat-inactivated fetal bovine serum (Gibco, FBS), in a humidified atmosphere of 5% CO_2_ at 37°C, until reaching 80% confluency. The medium was changed every 3 days. These cells were divided into four treatment groups.

### Animals and experimental procedures

Balb/c mice(4-6 weeks, weighing 21-23 g)and albino rabbits(weighing 1.5-2.5 kg) were provided by animals’ raising house of Shenyang Medical College (Certificate of Conformity: Liao [2010] No. 022). The experimental animals were housed separately at room temperature (20 ± 2)°C, humidity 55%-60%. They were provided a standard laboratory diet and water *ad libitum*. Before the experiments, we measured the body temperature of the rabbits twice a day for three consecutive days. The rabbits which temperature range from 38.6°C to 39.5°C, and which temperature fluctuation range less than 0.3°C were selected. All of the experimental procedures were carried out in accordance with the NIH Guidelines for the Care and Use of Laboratory Animals. The animal experiments were approved by the Institutional Animal Care and Use Committee of the Peking University.

The establishment of LT2-infected Balb/c mice model—All the Balb/C mice challenged via the oral administration with 7.6 × 10^5^ CFU LT2 by blunt-tipped gauge needle were divided into five groups. Each mice in berberine group was inoculated orally with 0.5 ml LB broth in the presence of berberine with different final concentrations (10, 20, 30, 40 mg/kg) for 7 consecutive day. While the mice in non-treatment group inoculated orally with 0.5 ml LB broth for consecutive 7 days was set as control. The survival of mice in each group was assessed once a day for 8 consecutive days.

The establishment of LPS-treated Balb/c mice model—Each Balb/c mouse was injected intraperitoneally with 2EU/ml bacterial endotoxin(0.5 ml for each mouse). The mice in berberine group were administered intragastrically with berberine solution at the dose of 0.20, 0.16 or 0.13 g/kg, in comparison with the non-treatment mice which were administered intragastrically with distilled water (0.01 ml/g). The survival of mice in each group was assessed once an hour throughout the experiment.

The establishment of LPS-treated pyretic rabbits model—Before the oral administration, the rectal temperature was measured once an hour for 2 hours, and the average value was set as the basal body temperature. The rabbits in berberine group were administered intragastrically with berberine solution at the doses of 0.06, 0.05 or 0.04 g/kg, in comparision with the non-treament rabbits. One hour after the administration, the rabbits were injected with 0.8 EU/kg bacterial endotoxin through their ear vein. Lastly, the body temperature was measured once an hour for five consecutive hour and the changes in body temperature (the difference between the body temperature and basal body temperature pyrogenic Δ T °C) were calculated.

### Docking studies with autodock 4.2

The crystal structure of human MD-2 complexed with lipid IVa (2E59. pdb) [37] was used for docking studies with Autodock v4.2. The protein was processed by removing the native ligand and addition of polar hydrogen atom and Kollman united-atom partial charges. The berberine was prepared by assigning Gasteiger–Marsilli atomic charge and defining active torsion groups. Grid maps were calculated with 40 × 86 × 54 points spacing by 0.375 Å, centered at point (0.445, 23.87, 13.078) using Autogrid4. The docking studies were carried out with flexible ligand using Lamarckian Genetic Algorithm with the following parameters: translation step (2 Å), quaternion step (50°), torsional step (50°), torsinal degree of freedom (2). All other parameters were set to their default values. The protein and ligand berberine were further prepared with AutodockTools [38]. The analysis of the docking was performed with AutoDockTools, and PyMOL software [39].

### Molecular dynamics simulations

Molecular dynamics (MD) simulations were carried out with Gromacs v4.5 program [40,41]. The structure of protein was taken from the Protein Data Bank (2E59.pdb). Gromos96 (53a) force field was used for the protein, and the parameters for the berberine are generated with PRODRG web server [42,43]. The protein was solvated with a rectangular water box extending 10 Å from the protein. Single Point Charge (SPC) model was used for the waters. Four berberine moleculars were randomly placed around the protein within 2 Å. Two Cl^-^ ions were added to neutralize the total charge of system.

In preparation for MD, the system was first subjected to 1000 steps of steepest descent minimization. Then the system was heated to the 298 K with the protein partially constrained. Finally 85 ns MD simulation for equilibration and production was carried out for the system with 3D periodic boundary in NPT ensemble imposed. Weak coupling methods [44] were applied with coupling constants of 0.1 ps and 0.5 ps respectively to maintain the systems at 298 K and 1 Bar. Bonds involving hydrogen were constained with LINCS algorithm for protein and SETTLE algorithm for water [45,46], thus permitted using a integrate step of 2 fs. Electrostatic interactions were treated by utilizing Particle Mesh Ewald (PME) method [47,48] beyond the cutoff at 10 Å. Van der Waals interactions were cut off at distance of 14 Å. Neighbor lists were used with a list cutoff of 10 Å and update frequency of every 10 steps. Snapshots of trajectories were saved every 10 picoseconds for analysis.

### Real-time RT-PCR analysis of NF-κB, IL-6, TNFα, and IFNβ mRNA expression

Total RNA was recovered from RAW264.7 cells using the Trizol reagent (Invitrogen) and Dnase treated using Turbo Dnase® (Ambion). RNA was reverse transcribed using the Superscript™ first-strand cDNA synthesis kit (Invitrogen). Primer sequences for mouse NF-κB were 5′-CAG CCA GGA TTG AGG ATA TGA G-3′ and 5′-TTC GGA CAA CAG AAG TCA GGA G-3′; for IL-6 were 5′-AAC GAT GAT GCA CTT GCA GA-3′ and 5′-GAG CAT TGG AAA TTG GGG TA-3′; for TNFα were 5′-TCC CCA AAG GGA TGA GAA GTT C-3′ and 5′-TCA TAC CAG GGT TTG AGC TCA G-3′; for IFNβ were 5′-CCA CAG CCC TCT CCA TCA AC-3′ and 5′-CTA CCA CCA GGC TCG TCT CT-3′; for β-actin were 5′-ATG GGT CAG AAG GAC TCC TAC G-3′ and 5′-AGT GGT ACG ACC AGA GGC ATA C-3′, for the sense and antisense primers, repectively. Thermal cycling conditions for the PCR reactions were 94°C for 5 min followed by 40 cycles of 94°C for 45 s, 61°C for 30 s, and 72°C for 30 s. Polymerase reaction products amplified by these primers were cloned into pGEMT vector (Promega) and sequenced for verification. Real-time reactions were carried out on a real-time machine (Biorad) using the IQ™ SYBR® Green Supermix kit (Biorad). The abundance of each gene product was calculated by regressing against the standard curve generated in the same reaction with their respective plasmids. The NF-κB, IL-6, TNFα, and IFNβ values for each sample were normalized to β-actin.

### Statistical methods

Statistic analyses were performed by using SPSS 13.0 software. The data are expressed as mean ± SD. Student’s T test was used in the data statistics. Values of P < 0.05 were considered significant.

## Results

### Anti-bacterial effects of berberine

**Figure 1:**
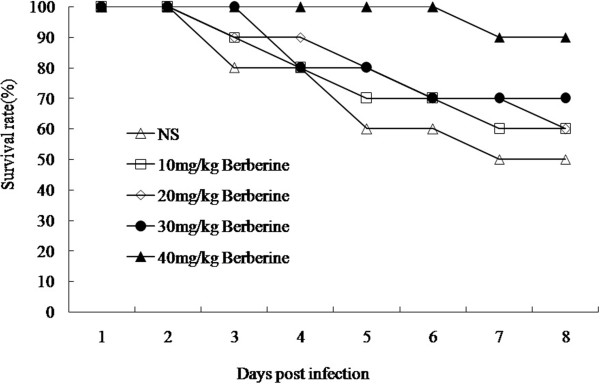
**Therapeutic effect of berberine on LT2-challenged Balb/c mice.** n = 10 in each group. Each mouse was challenged with 7.6 × 10^5^ CFU LT2. Mice in berberine group was inoculated orally with 0.5 ml LB broth in the presence of berberine with different final concentrations (10, 20, 30, 40 mg/kg) for consecutive 7 days, whereas mice in control group was inoculated orally with 0.5 ml LB for consecutive 7 days. The survival was assessed every 24 h throughout the experiment.

As described previously, berberine has been widely used for treatment of bacterial infection disease, and presents antibacterial acitivity. However, the role of berberine in antibacterial action has not been extensively studied. Therefore, we first investigated its effect on LT2 infected mice. Preliminary test have presented the LD50 of Salmonella typhimurium LT2 gavage at 7.5 × 10^5^ CFU per mouse and each 4-6-week old Balb/c mouse was challenged with LD50 of LT2. The results show that 50% of the mice without berberine treatment died by the end of the 8th day following infection. By contrast, after treated with 10 mg/kg, 20 mg/kg, 30 mg/kg and 40 mg/kg berberine, 60, 60, 70 and 90 percent of the infected mice survived to the 8th day, respectively (Figure 1). Meanwhile, the infected mice without berberine treatment lost 18.96 ± 2.58 percent of their body weight, whereas those in the berberine treatment group maintained or increased their weight. Meanwhile, we also proved that berberine have no effects in bacteriostasis (data not shown).

### Effects of berberine on the survival from LPS induced death of BalB/C mouse

**Figure 2:**
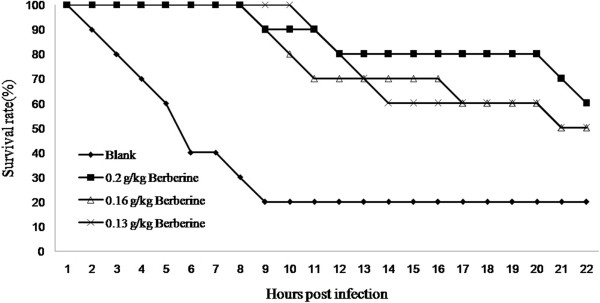
**Therapeutic effect of berberine on LPS-challenged Balb/c mice.** n = 10 in each group. Each mouse was injected with 2 EU/ml bacterial endotoxin (0.5 ml for each). The LPS-challenged mice was administered intragastrically with distilled water (0.01 ml/g) or berberine at doses of 0.20, 0.16 or 0.13 g/kg. The survival was assessed once an hour throughout the experiment. The average death time of each group was calculated within Mean ± SD adopted. P < 0.05 vs control.

It is known that endotoxin(LPS) plays an important role in the bacterial infection process and berberine was expected to present some anti-endotoxin effects. Therefore, LPS-challenged Balb/c mice model was established to investigate whether berberine could reduce the mortality rate of mice challenged with LPS. Preliminary test indicated that the mice injected intraperitoneally with 2 EU/ml endotoxin solution (0.5 ml of each) reached a lethal rate of 80% and every 4-6-week old Balb/c mouse were injected with 2 EU/ml bacterial endotoxin(0.5 ml for each mouse). The results show that 80% of the mice without berberine treatment died, and the average death time is 6.27 ± 4.72 hours. By contrast, After treated with 0.20 g/kg, 0.16 g/kg and 0.13 g/kg berberine, 60, 50, and 50 percent of the infected mice survived respectively, and the average death time of each group were 16.17 ± 7.77, 14.89 ± 5.74 and 12.96 ± 6.51 hours, which was significantly better than that of mice only exposed to LPS (P < 0.05) (Figure 2).

### Effects of berberine on the LPS induced fever of pyretic rabbit

**Figure 3:**
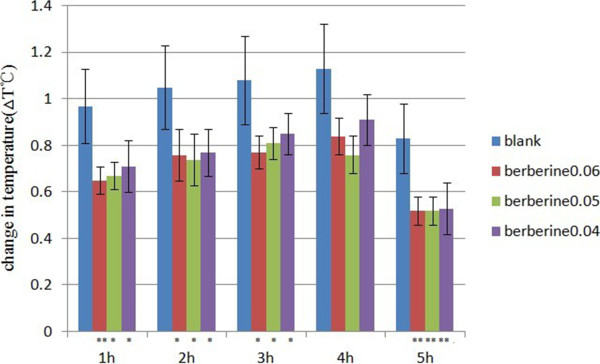
**Changes in body temperature of the LPS-challenged rabbits.** n = 6 in each group. The rabbits were administered with berberine at the doses of 0.06, 0.05 or 0.04 g/kg, in compartion with the non-treament rabbits. Each rabbit was injected with 0.8 EU/kg LPS. The changes in body temperature was assessed every 24 h throughout the experiment. Mean ± SD. ^**^P < 0.01, ^*^P < 0.05 vs control.

Based on the hypotheis that berberine may block the LPS signaling, berberine is expected to present the ability to relieve fever caused by LPS. In the present study, we investigated the antipyretic effect of berberine on LPS-challenged rabbits. Each rabbit was injected with 0.8 EU/kg LPS. The changes in body temperature of the rabbits treated with 0.06, 0.05 or 0.04 g/kg berberine are significantly lower than those of the non-treatment group, and the 0.05 g/kg treatment group presented the most siginificant effects. (P < 0.05) (Figure 3).

### Role of berberine in the combination between LPS and its receptor molecules

**Figure 4:**
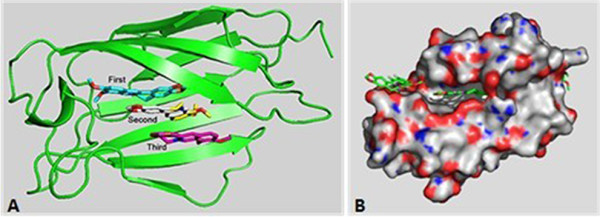
**Role of berberine in the combination between LPS and its receptor molecules. A**. The docking analysis of berberine and MD-2. The binding free energies of the three berberine molecules was 7.70 kcal/mol, -7.33 and -6.75 kcal/mol respectively. **B**. A MD simulation of a system with four molecules of berberine randomly distributed around the MD-2 solvated by water.

As described above, berberine exhibits significant anti-endotoxin effects. To gain insights into the possible antibacterial mechanism, we further investigated the role of berberine LPS/TLR4 signaling pathway.The LPS triggers signal transduction pathway leading to inflammatory reaction by binding to TLR4/MD-2 receptor via hydrophobic interaction. Being characteristized by its hydrophobic surface, berberine was expected to exibit an antibacterial activity by acting as an LPS antagonist. To prove this assumption, we carried out docking calculation to explore the interaction between berberine and MD-2. Our docking study demonstrated that berberine could bind to free MD-2 with binding free energy of 7.70 kcal/mol. The binding cavity of MD-2 is large enough that it would still have sufficient empty space even though a molecule of berberine had bound to it. To investigate whether the MD-2 could hold more than one berberine. we docked berberine into MD-2 continually. As a result, the cavity of MD-2 could hold two more berberine molecules with the binding energies of -7.33 and -6.75 kcal/mol respectively. Therefore, a MD-2 receptor with an open cavity could accept at least three berberine molecules. And the molecular planes, were arrangred nearly in parallel (Figure 4A).Since proteins are usually solvated by water under physiological conditions, it is essential to investigate whether berberine could bind to MD-2 under the influence of water solvent. Therefore, we carried out a MD simulation of a system with four molecules of berberine randomly distributed around MD-2. The initial construction was subtracted from the crystal structure (2E59.pdb) with an open cavity. And we found that the presence of water solvent squeezed MD-2 so strongly that the hydrophobic cavity of the MD-2 was collapsed through MD simulation. However, one of the four molecules of berberine was still observed to enter the cavity of MD-2 after 85 ns of MD simulation (Figure 4B). Meanwhile, another molecule of berberine approached to the enrance to the cavity.

### Role of berberine in LPS induced expression of NF-κB and cytokines

**Figure 5:**
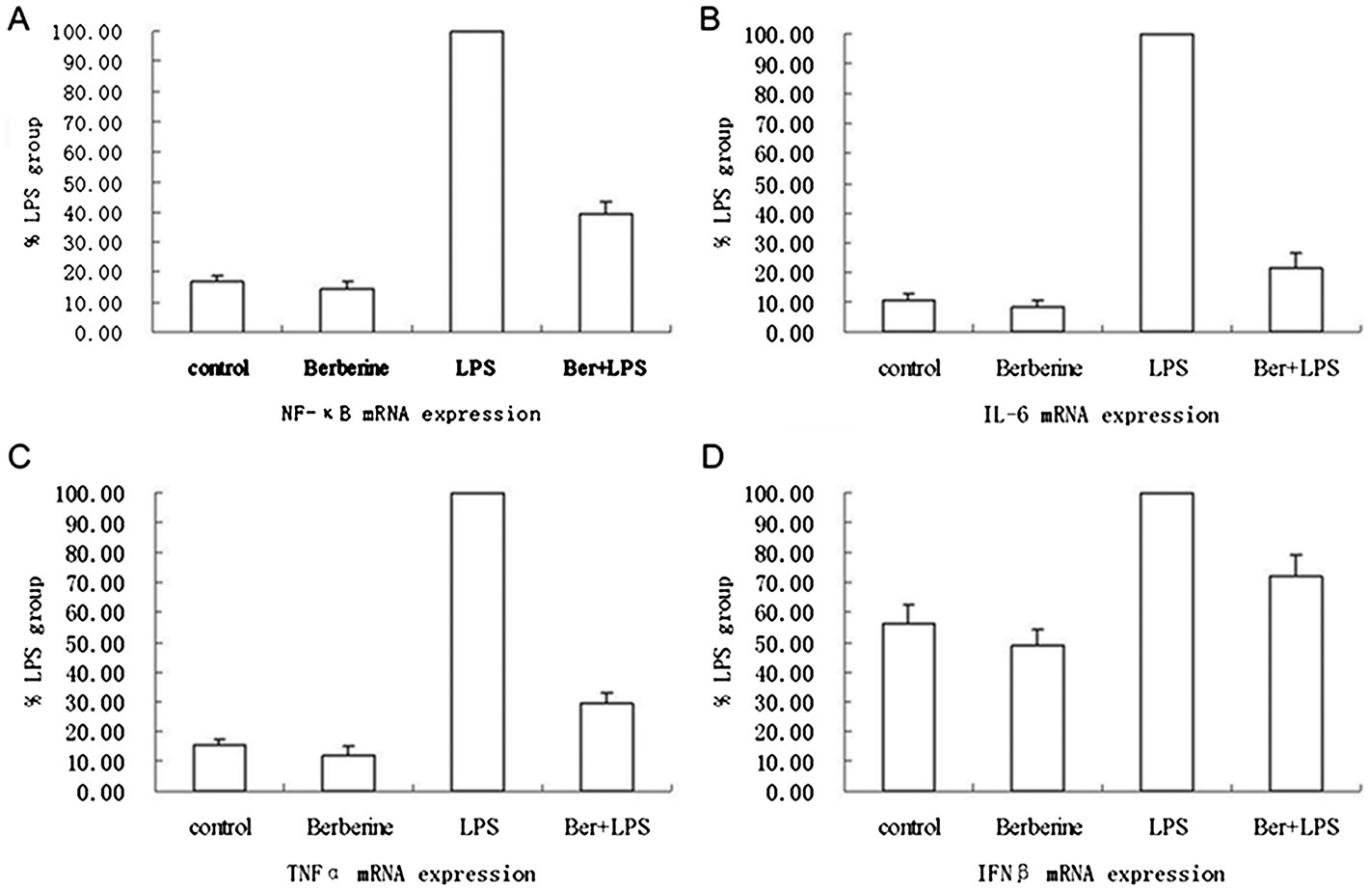
**The Realtime PCR analysis for NF-κB (A), IL-6 (B), TNFα and IFNβ mRNA expression in RAW264.7.** It was conducted 1 h after treatment as described previously. LPS group was recognized as a control and data was expressed as the ratio to LPS group. **A**, **B**, **C** and **D** showed the expression of NF-κB, IL-6, TNFα and IFNβ, respectively.Each group was treated with NS(control), berberine, LPS and LPS mixed with berberine, respectively.

Upon challenge with LPS, cells form a broad spectrum of immune mediators such as cytokines which can lead to LPS-related disease states. To confirm that berberine could block the LPS signaling, we employed real-time analysis in the present study. we proved that berberine could significantly inhibit the increasing expression of NF-κB, IL-6, TNFα and IFNβ stimulated by LPS in the LPS-challenged RAW264.7, in comparison with the negative control (Figure 5).

## Discussion

Berberine has been used in the treatment of enteritis [5,6] for thousands of years in China, and presents the advantages of shorter treatment course, faster currative effect, little side effect and milder tolerance. However, the mechanism of berberine in anti-bacterial is still unclear.

In this study, we showed that berberine had no effect on bacteriostasis. As known, LPS plays an important role in bacterial infection. Therefore, we assumed that the anti-bacterial effects of berberine might be related to its inhibitory action on LPS infection. As we expected, The results indicated that berberine could reduce the lethal rate of mice infected by LPS, and extend the survival time. Subsequently, we demonstrated that berberine could significantly relieve fever of rabbits induced by LPS. Based on these, we believe that there must exsits some definite link between berberine and LPS signaling.

The real-time pcr analysis demonstrated that berberine can reduce the mRNA expression of NF-κB, IL-6, TNFα and IFNβ in LPS-challenged RAW264.7. Other studies have indicated that LPS significantly induced enterocyte apoptosis, increased TLR4 mRNA expression, MIP-2 production, I-κBα phosphorylation, and myeloperoxidase content in the ileum and pretreatment of berberine alleviated all the alterations [14]. Beside these, another study showed that berberine can suppress the activation of TLR4 and NF-κB in the ileum of mice [15]. Those mentioned studies seem to verify that berberine can block the LPS/TLR4 signaling pathway. But former studies have not revealed how berberine block the signaling.

Since the binding of Lipid A and the hydrophobic cavity of MD-2 play an key role in LPS recognition, and a berberine molecule has a large hydrophobic surface, we hypothesized that berberine would be likely to compete to bind TLR4/MD-2, thereby blocking the signaling. We first adopt docking calculation to investigate whether the berberine molecules can bind to the MD2. The results indicated that berberine could bind to free MD-2 with binding free energy of 7.70 kcal/mol. The binding cavity of MD-2 is large enough that it would still have sufficient empty space although a molecule of berberine have already bound to it. Afterward, the other berberine molecules was docked continually into MD-2. As a result, the cavity of MD-2 could hold two more berberine molecules with the binding energies of -7.33 and -6.75 kcal/mol respectively. Therefore, a MD-2 receptor with an open cavity could accept at least three berberine molecules. Furthermore, to investigate whether berberine could bind to MD-2 under the influence of water solvent, a MD simulation of a system with four molecules of berberine randomly distributed around MD-2 was carried out. The presence of water solvent squeezed MD-2 so strongly that the hydrophobic cavity of the MD-2 was collapsed. However, one of the four molecules of berberine was still observed to enter the cavity of MD-2 after 85 ns of MD simulation. Meanwhile, another molecule of berberine approached to the enrance to the cavity. It can be expected that this molecule might also enter the pocket if a longer simulation was performed.

**Figure 6:**
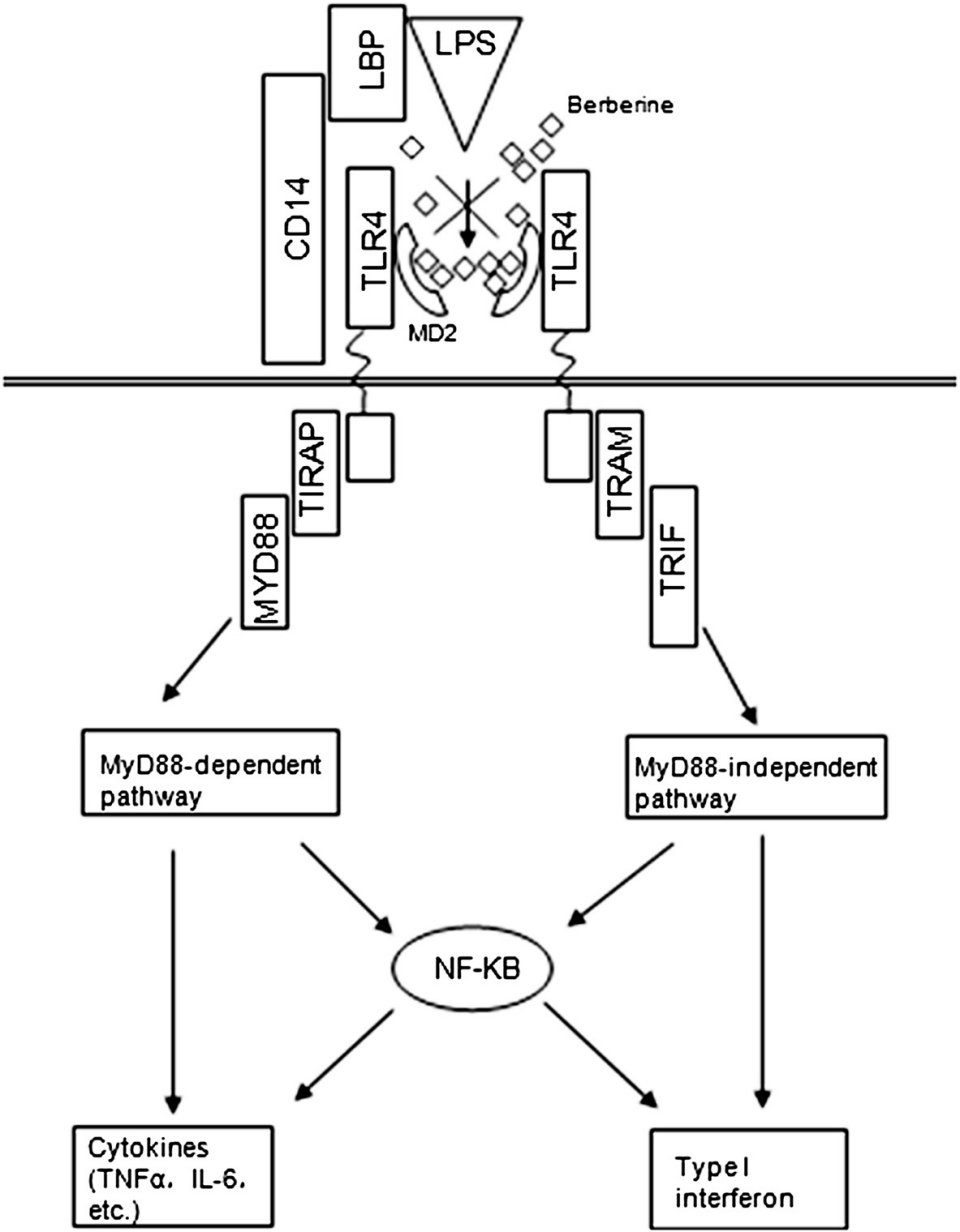
**The role of berberine in TLR4/MD-2 signaling.** The recognition of LPS is facilitated by LBP and CD14, and is mediated by TLR4/MD-2 receptor complex. Upon the recognition, LPS/TLR4 signaling can be separated into MyD88-dependent and MyD88- independent pathways, which mediate the activation of proinflammatory cytokine and Type I interferon genes.

As it was mentioned above, the compression of water solvent rendered the cavity of MD-2 narrow down and the lipid A of LPS is too large to get into the narrow cavity directly. Therefore, some transitional proteins are necessary. The induction of inflammatory responses by LPS is achieved by the coordinated and sequential action of four principal endotoxin-binding proteins: lipopolysaccharide-binding protein (LBP: a soluble shuttle protein which directly binds to LPS and facilitates the association between LPS and CD14), CD14 (a glycosylphosphatidylinositol-anchored protein which facilitates the transfer of LPS to the TLR4/MD-2 heterodimers and modulates LPS recognition), MD-2 and TLR4 [49-53]. By contrast, berberine do not generally require the canonical LPS presentation sequence to bind the receptor. As the size of molecule is small enough, berberine molecules can directly bind to MD-2 with the hydrophobic surface. From this, we can easily speculate that the more berberine molecules get into the cavity, the smaller chance that LPS can bind to MD-2, which suggest that berberine take priority over LPS in binding MD-2, thus interrupting the recognition of LPS. Overall, we provided a theoretical basis that berberine can act as a receptor antagonist— it can block the LPS/TLR4 signaling pathway from the sourse (Figure 6) and prevent the immune response induced by LPS.

TLR4 has therefore been recognized as an important pharmacological target. In addition to being responsible for fever and diarrhea, the TLR4 activation caused by endotoxin with subsequent cytokine production can lead to life-threatening syndromes such as sepsis and septic shock. Mortality of patients who has suffered from septic shock is still 40%–60% despite rapid progress in developing antibiotics and other therapeutic methods in clinical practice [12]. Some drugs were designed and synthesized to interrupt LPS from binding to TLR4/MD2 receptor complex, such as Eritoran, Taxol and some protein drugs. With the advantage of lower cost, faster currative effect, and little side effect, berberine, the newly-discovered LPS antagonist, could be widely utilized as a substitute for expensive drugs in the treatment of LPS-induced diseases.

Furthermore, in recent years, the phenomenon of the over prescription ofantibiotics have led to increased bacterial resistance to antibiotics. Berberine are expected to be such a kind of drug which can be widely used in the clinical treatment of wide range of diseases instead of antibiotics in order to decrease the over use of antibiotics which have led to progression of increased bacterial antibiotic resistance.. Based on this, it is essential to figure out the antibacterial mechanism of berberine in order to provide a theoretical basis for the further application of berberine in the treatment of diseases caused by the infection of bacteria and the endotoxin (LPS) released by bacteria. Since the process of bacterial infection is complicated, there might exist other possible antibacterial mechanisms of berberine which we will further investigate. We believe that further study on berberine will aid in our ability to design effective interventions and treatments for bacterial infection diseases.

## Conclusion

Berberine can act as a LPS antagonist and block the LPS/TLR4 signaling from the sourse, resulting in the anti-bacterial action.

## Competing interests

The authors declare that they have no competing interests.

## Authors’ contributions

RD conceived the study, carried out the molecular dynamics simulations, the docking analysis and the animal experiments, performed the statistical analysis, and drafted the manuscript. MC conceived the study, performed Real-time RT-PCR analysis, and revised the manuscript. XYL and SHX performed the cell cuture process. ZYC performed the animals experiments and participated in the design of the study. MBZ participated in the design of the study, performed the docking analysis as well as the molecular dynamics simulations. YJZ helped to drafted the manuscript. YW participated in the design of the study and revised the manuscript. All authors read and approved the final manuscript.

## Pre-publication history

The pre-publication history for this paper can be accessed here:

http://www.biomedcentral.com/1472-6882/14/89/prepub
